# The complete mitochondrial genome of *Sarcophaga pterygota* (Diptera: Sarcophagidae)

**DOI:** 10.1080/23802359.2019.1660272

**Published:** 2019-09-06

**Authors:** Shiwen Wang, Yanjie Shang, Lipin Ren, Li Yang, Yadong Guo

**Affiliations:** a Department of Forensic Science, School of Basic Medical Sciences, Xinjiang Medical University, Ürümqi, China;; b Department of Forensic Science, School of Basic Medical Sciences, Central South University, Changsha, Hunan, China

**Keywords:** Mitochondrial genome, *Sarcophaga pterygota*, phylogenetic relationships

## Abstract

*Sarcophaga pterygota* (Diptera: Sarcophagidae) plays a crucial role in medical and veterinary management. The complete mitochondrial genome (mitogenome) of *S. pterygota* was first sequenced and annotated. The circle DNA is composed of 13 protein-coding genes (13 PCGs), 2 ribosomal RNA (2 rRNAs), 22 transfer RNA (22 tRNAs) and a AT-rich region. It shows that arrangement of the genes is similar with the classical metazoan. The size of mitogenome is 15,236 bp containing A (40.0%), G (9.3%), T (36.6%), and C (14.1%). Moreover, phylogenetic analysis reveals that the branch of *S. pterygota* is clustered separately. This study enriches the mitogenome database of flesh flies and represents progress for analyzing of phylogenetic relationships.

*Sarcophaga pterygota* Thomas 1949 was commonly found on decomposed carcasses or garbage, which may play a crucial role in medical and veterinary management (Thomas 1949; Pérez-Moreno et al. 2006). In recent years, molecular approach has been proved to be a suitable tool in species identification of insects and that mitogenome was thought to be effective biological markers (Renaud et al. 2012; Shang et al. 2019). In this study, the circle DNA is composed of 13 protein-coding genes (13 PCGs), 2 ribosomal RNA (2 rRNAs), 22 transfer RNA (22 tRNAs) and a AT-rich region. The size of mitogenome is 15,236 bp, containing A (40.0%), G (9.3%), T (36.6%), and C (14.1%) (Genbank No. MK820722).

Adult specimens of *S. pterygota* were trapped in Beijing, China (39°26′N; 115°25′E) in May 2017. All of these specimens were identified morphologically under the microscope by an expert and then preserved in Guo’s Laboratory (Changsha, Hunan, China) with a sole code (CSU19040904). DNA was extracted from thoracic tissues of each adult fly by using the QIANamp Micro DNA Kit (TIANGEN BIOTECH CO., LTD). Subsequently, sequences were conducted on an Illumina HiSeq 2500 Platform (Ren et al. 2019). The remaining samples were stored at −80 °C.

Phylogenetic analysis of *S. pterygota* and 11 sarcophagid species was performed based on 13PCGs using neighbor-joining (NJ) inference method, including two blow flies as an outgroup (Figure 1). Phylogenetic analysis reveals that *S. pterygota* belongs to the *Pierretia* subgenus, which separately diverges from the clade of Sarcophagidae species. This study contributes useful information for further investigation on phylogeny as well as species identification.

**Figure 1.**
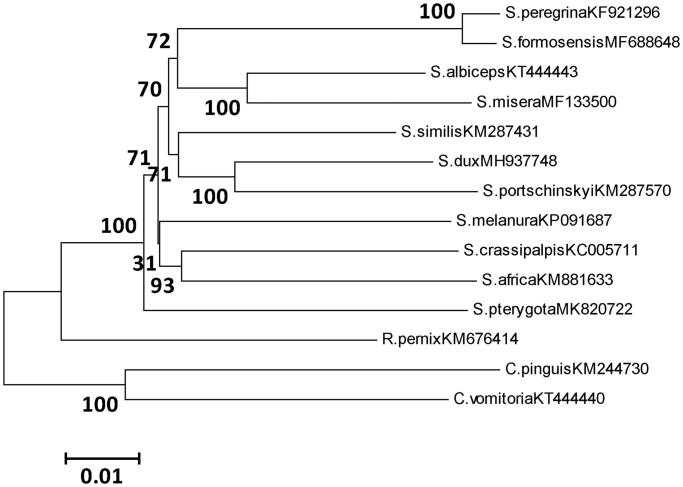
Topology from the NJ method of phylogeny for 12 sarcophagid species based on 13PCGs. Morphological species identifications were assigned for all specimens along with voucher IDs. Outgroups were *Calliphora* specimens: *Calliphora vomitoria* (KT444440), *Chrysomya pinguis* (KM244730). Evolutionary distance divergence scale bar was 0.01.
